# Successful treatment of an elderly patient with relapsed/refractory angioimmunoblastic T-cell lymphoma with the PI3Kδ inhibitor linperlisib: a Case Report

**DOI:** 10.3389/fphar.2025.1554501

**Published:** 2025-05-15

**Authors:** Ming-Qiang Chu, Ting-Juan Zhang, Qian Yang, Yuan Feng, Chao Lu, Yong-Hui Ji, Jun Qian, Jing-Dong Zhou

**Affiliations:** ^1^ Department of Hematology, The Affiliated People’s Hospital of Jiangsu University, Zhenjiang, Jiangsu, China; ^2^ Institute of Hematology, Jiangsu University, Zhenjiang, Jiangsu, China; ^3^ Zhenjiang Clinical Research Center of Hematology, Zhenjiang, Jiangsu, China; ^4^ The Key Lab of Precision Diagnosis and Treatment of Zhenjiang City, Zhenjiang, Jiangsu, China; ^5^ Department of Oncology, The Affiliated People’s Hospital of Jiangsu University, Zhenjiang, Jiangsu, China; ^6^ Department of Medical Imaging, The Affiliated People’s Hospital of Jiangsu University, Zhenjiang, Jiangsu, China

**Keywords:** angioimmunoblastic T-cell lymphoma, relapsed/refractory, PI3Kδ inhibitor, linperlisib, elderly patients, salvage therapy, case report

## Abstract

Angioimmunoblastic T-cell lymphoma (AITL), a highly aggressive peripheral T-cell lymphoma (PTCL), carries a poor prognosis in elderly patients due to frequent relapse and limited salvage options after multiline therapy. We present the case of an 80-year-old woman with relapsed/refractory (R/R) AITL who relapsed after CHOP and exhibited resistance to the following sequential therapies: second-line chidamide plus COP and third-line chidamide with mitoxantrone hydrochloride liposome. Molecular analysis revealed *DNMT3A* and *IDH2* mutations, reflecting disease complexity. Salvage therapy with linperlisib, a selective PI3Kδ inhibitor, combined with gemcitabine/oxaliplatin induced sustained partial remission, followed by linperlisib maintenance. The regimen demonstrated exceptional safety, with no grade ≥2 toxicities, even in this frail population. This case highlights the dual role of linperlisib as an effective and well-tolerated therapy for elderly R/R AITL patients who have exhausted prior lines. By precisely targeting PI3Kδ, our findings offer critical real-world evidence to address the unmet need for safe salvage strategies in this vulnerable population.

## Introduction

Angioimmunoblastic T-cell lymphoma (AITL), reclassified as nodal TFH cell lymphoma, angioimmunoblastic type (nTFHL-AI) in the 2022 WHO-HAEM5 guidelines (Alaggio et al., 2022), is a highly aggressive peripheral T-cell lymphoma (PTCL) that originates from follicular helper T (Tfh) cells (de Leval et al., 2007; Dupuis et al., 2006). As one of the most prevalent PTCL subtypes globally (de Leval et al., 2015; Vose et al., 2008), AITL is characterized by mutations in the epigenetic regulatory genes (*TET2*, *DNMT3A*, and *IDH2* ^*R172*^), T-cell receptor-related genes, and the pathognomonic *RHOA* ^*G17V*^ mutation (Chiba and Sakata-Yanagimoto, 2020; Willemsen et al., 2018). These genetic alterations synergize with the tumor microenvironment, which is enriched in dendritic cells, macrophages, T cells, and B cells, driving therapeutic resistance and aggressive disease progression (Chiba and Sakata-Yanagimoto, 2020).

Clinically, AITL predominantly affects elderly patients (median age ≥60 years), with over 50% presenting with advanced-stage disease accompanied by B symptoms, which include unexplained fever (≥38°C), drenching night sweats, and unintentional weight loss (>10% of body weight within 6 months) (Advani et al., 2021; Wei et al., 2023; Xu and Liu, 2014; Yoon et al., 2021). Despite CHOP-based frontline therapy, outcomes remain dire. The International T-cell Project reported a 5-year progression-free survival (PFS) of 13% and a 5-year overall survival (OS) of 21% for high-risk AITL score patients (Advani et al., 2021; Mohammed Saleh et al., 2021). Although hematopoietic stem cell transplantation offers curative potential for a minority (13% of eligible patients), it is rarely feasible in the elderly or frail populations, leaving salvage therapies as the primary option (Advani et al., 2021).

The prognosis of relapsed/refractory (R/R) AITL is catastrophic, with a median survival of <2 years after relapse (Advani et al., 2021). Elderly patients face compounded challenges owing to cumulative toxicity and intolerance to conventional regimens. Although novel agents such as HDAC inhibitors and bispecific antibodies have demonstrated modest efficacy, their toxicity limits their utility in vulnerable elderly cohorts (Frutos Díaz-Alejo et al., 2024). Linperlisib, a selective PI3Kδ inhibitor, has emerged as a promising candidate. By targeting PI3Kδ, linperlisib combines mechanistic precision with a favorable safety profile, as evidenced in clinical trials (Jiang et al., 2021; Zou et al., 2022).

We present the case of an 80-year-old woman with R/R AITL who relapsed after CHOP therapy and developed resistance to the following sequential therapies: second-line chidamide + COP (Chi + COP) and third-line chidamide + mitoxantrone hydrochloride liposome (Chi + Lipo-MIT). Salvage therapy with linperlisib combined with gemcitabine/oxaliplatin (GemOx) induced sustained partial remission, followed by maintenance monotherapy with no grade ≥2 toxicities. This case highlights the dual efficacy and tolerability of linperlisib in elderly R/R AITL patients, a population historically underserved by existing therapies, and it provides a critical real-world validation for its role in addressing this clinical gap.

## Case presentation

**FIGURE 1:**
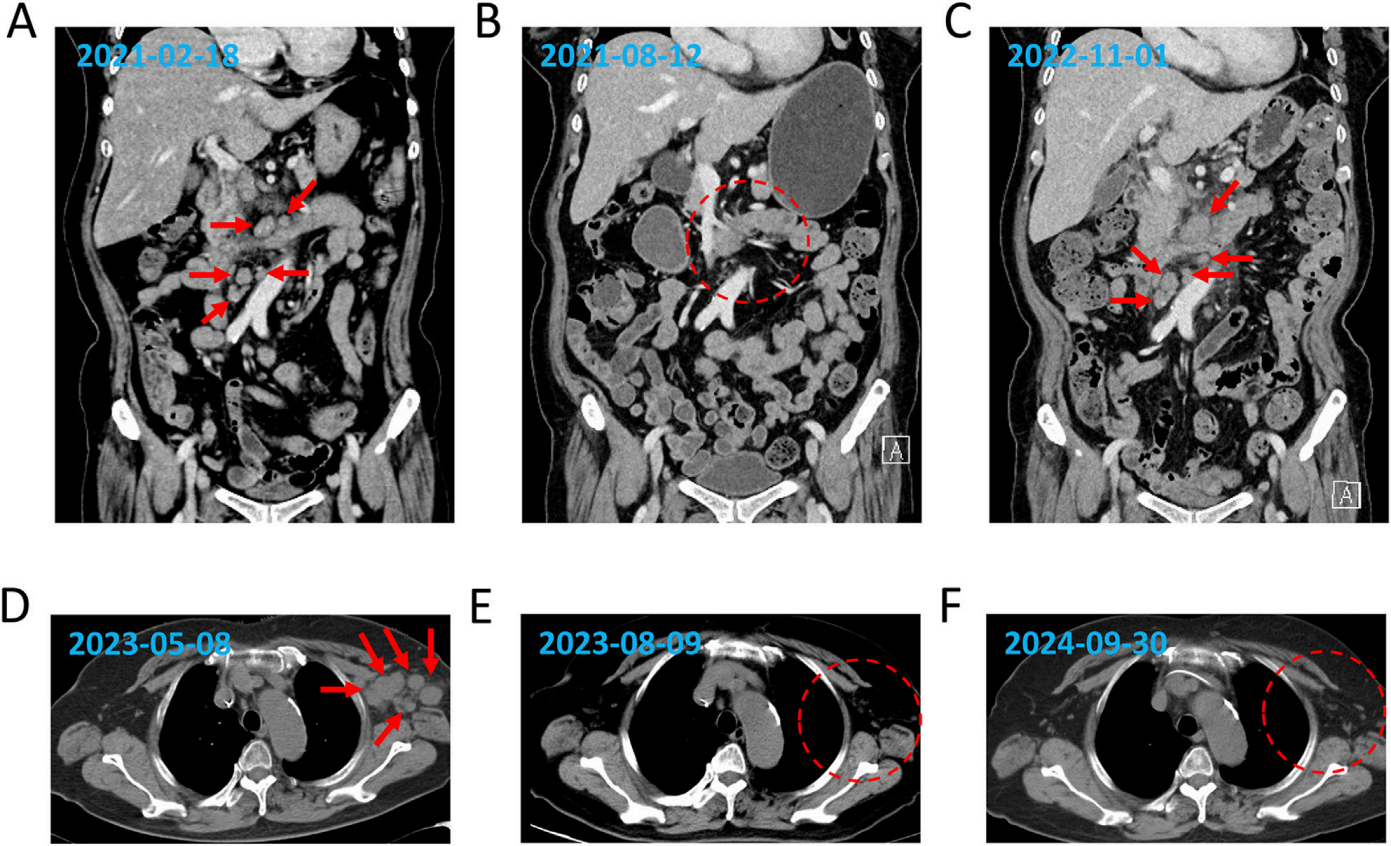
Dynamic evaluation of enlarged lymph nodes using CT. Enlargement and reduction of lymph nodes at initial diagnosis **(A)**, PR1 **(B)**, relapse **(C)**, refractory **(D)**, PR2 **(E)**, and SD **(F)** by CT evaluation. The red arrows indicate the enlarged lymph nodes, and the red dotted frames represent the reduction of the enlarged lymph nodes.

A 78-year-old Chinese woman presented to our hospital on 18 February 2021 with a 3-day history of bloating and fever for 1 day. The patient had no significant medical or family history of malignancy, autoimmune disorders, or hereditary diseases. Psychosocial assessment revealed that the patient was a farmer with no history of smoking, alcohol use, or psychotropic medication. Physical examination revealed enlargement of the bilateral axillary lymph nodes along with mild pressure pain in the mid-upper and right-lower abdomen. Laboratory findings included slightly reduced serum albumin (ALB, 32 g/L), elevated liver enzymes [alanine aminotransferase (ALT), 58 U/L; alkaline phosphatase (ALP), 136 U/L; and gamma-glutamyl transferase (GGT), 83 U/L], and elevated C-reactive protein (CRP: 97.39 mg/L). Lactate dehydrogenase (LDH) and peripheral blood counts [white blood cells (WBCs), platelets (PLTs), and hemoglobin (Hb)] were within the normal ranges. Epstein–Barr virus (EBV)-DNA testing was positive (20,000 copies/mL). Chest and abdominal computed tomography (CT) (Figure 1A) revealed multiple enlarged lymph nodes in the paracervical, supraclavicular, axillary, inguinal, intraperitoneal, and retroperitoneal regions. Positron emission tomography–computed tomography (PET–CT) showed high uptake of ^18^F-fluorodeoxyglucose in multiple lymphadenopathies on both sides of the mediastinum (SUVmax 12.9) and as in the spleen, sternum, multiple vertebrae, nasopharyngeal region, and tonsillar soft tissue. Flow cytometry revealed 74.8% lymphocytes in the lymph nodes with unremarkable antigen expression. Lymph node pathology confirmed AITL. Immunohistochemical (IHC) staining of the left axillary lymph node revealed the following profile for lymphoma cells: CD20 (background B-cells +), CD3 (+), CD5 (+), CD43 (+), PD-1 (+), CXCL13 (+), CD21 and CD23 (resident FDC net +), CD30 (scattered isolated +), BCL6 (background cells +), BCL2 (+), CD10 (minority +), and Ki67 (+). IHC findings from the left inguinal lymph node were as follows: CD20 (background cells +), PAX5 (background cells +), CD3 (+), CD2 (+), CD5 (+), CD4>CD8, CD7 (+), PD-1 (+), CXCL13 (+), BCL2 (+), BCL6 (background +), CD10 (scattered +), MUM1 (background +), CD21 and CD23 (resident FDC net +), CyclinD1 (−), ALK (−), CD68 (scattered +), CD163 (scattered +), CD56 (−), GranB (+), TIA1 (+), PF (−), and Ki67(+). EBV-encoded RNA (EBER) by the *in situ* hybridization test was negative in the lymphoma cells. Bone marrow (BM) examination revealed no lymphoma infiltration. Ultimately, the patient was diagnosed with AITL (stage IV, group B, PIT 2, ECOG 2). She received eight cycles of CHOP therapy (cyclophosphamide 750 mg/m^2^ day 1, pirarubicin 50 mg/m^2^ day 1, vincristine 1.4 mg/m^2^ day 1, and prednisone 100 mg days 1–5) starting on 21 April 2021. PET–CT (28 June 2021) demonstrated partial reduction of lymphadenopathy, and follow-up CT (12 August 2021) confirmed significant lymph node regression (Figure 1B) and partial remission (PR). Post-treatment assessment confirmed sustained PR. Maintenance therapy with lenalidomide (25 mg daily) was initiated but discontinued because of disease relapse at 18 months (October 2022).

The patient was re-admitted to the hospital with a fever on 24 October 2022. Laboratory investigations revealed reduced serum ALB (32.9 g/L) and elevated ALP (220 U/L) and GGT (67 U/L) levels, whereas ALT and LDH levels remained within normal ranges. The CRP level was elevated (87 mg/L). Peripheral blood tests indicated a mild reduction in WBCs (3.7 × 10^9^/mL) and PLTs (87 × 10^9^/mL) counts, with Hb within normal limits. A BM smear revealed a small population of lymphoma cells, and immunophenotyping identified 1.1% monoclonal T lymphocytes. The IHC results for BM were as follows: PD-1 (−), CD3 (minority +), CD7 (minority +), CD20 (minority +), and BCL2 (occasionally +). Chest and abdominal CT (1 November 2022) revealed progressive lymphadenopathy compared to prior imaging (Figure 1C). The *DNMT3A* ^*R882*^ mutation was identified in both peripheral blood mononuclear cells (PBMCs) and bone marrow mononuclear cells (BMMCs) by digital droplet PCR with variant allele frequencies (VAFs) of 24.684% and 6.966%, respectively. The *IDH2* ^*R172*^ mutation was identified in BMMCs with a VAF of 2.624%, whereas no *IDH2* ^*R172*^ mutation was detected in PBMCs.

**FIGURE 2:**
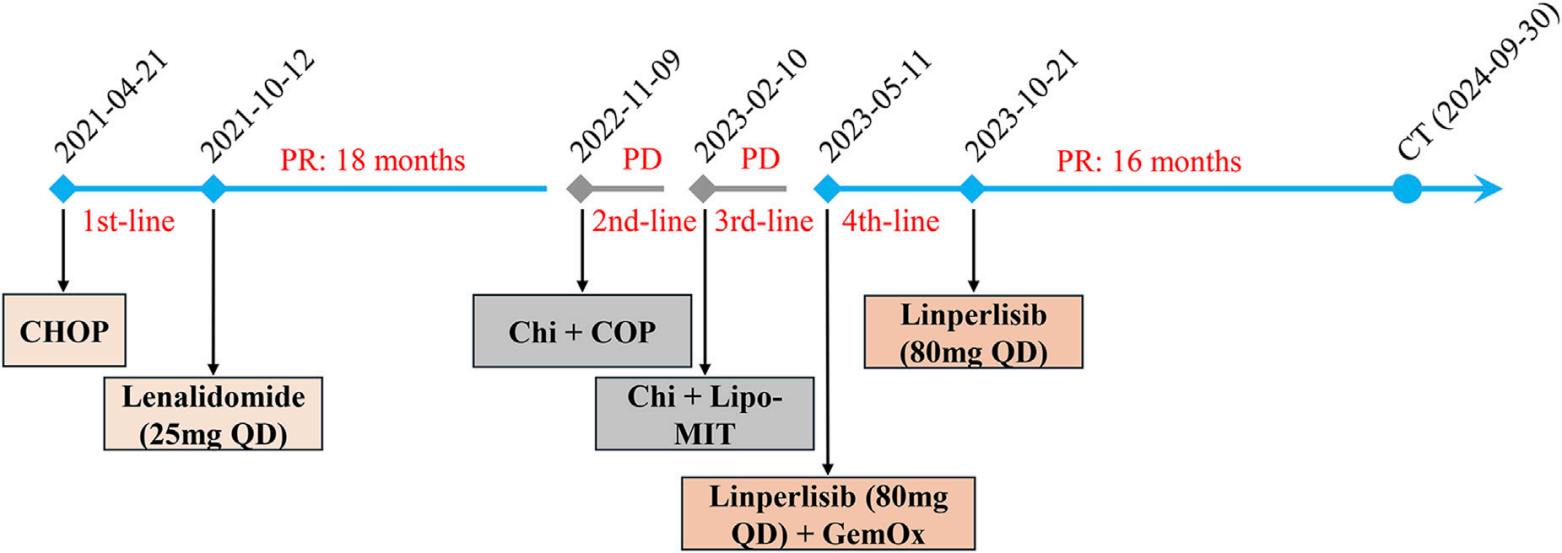
Timeline of the treatment process in this case.

The patient received two cycles of COP plus chidamide starting on 9 November 2022. Post-treatment CT confirmed progressive disease (PD) with further lymph node enlargement. Subsequently, the patient was treated with mitoxantrone hydrochloride liposome (Lipo-MIT); however, follow-up CT imaging (Figure 1D) demonstrated continued disease progression, confirming PD. Due to refractory disease after third-line therapy, salvage treatment with a novel regimen, linperlisib (a PI3Kδ inhibitor) (80 mg daily) combined with gemcitabine/oxaliplatin (GemOx), was initiated on 11 May 2023. Post-treatment CT revealed a marked reduction in lymphadenopathy, achieving PR (Figure 1E). The patient transitioned to linperlisib maintenance therapy, which has continued to date. The FACT-Lym total score of this patient improved from 82 at baseline to 112 at the 6-month follow-up (out of a maximum score of 148). The lymphoma-specific subscale demonstrated the most significant improvement (+15 points), indicating a marked alleviation of B symptoms. The most recent CT scan (Figure 1F) showed no evidence of abnormal lymphadenopathy. The treatment timeline is shown in Figure 2.

## Discussion and conclusion

The poor prognosis of R/R AITL highlights the urgent need for novel therapeutic strategies. These include the incorporation of multiple novel agents, such as HDACi, anti-CD30, anti-PD-1, enhancer of zeste 2 (EZH2) inhibitors, and farnesyltransferase inhibitors, into the treatment regimen. Chidamide is a benzamide class HDACi, and Lipo-MIT is a nanodrug containing mitoxantrone. Both have been approved by the China National Medical Products Administration for R/R PTCL. A favorable response to chidamide or Lipo-MIT has been observed in R/R AITL patients, with an overall response rate (ORR) of 50% (Gao et al., 2020; Huang et al., 2019; Shi et al., 2015). This included a response duration exceeding 34 months in three patients following treatment with chidamide (Shi et al., 2015). However, these trials enrolled only limited R/R AITL cohorts, necessitating validation in larger studies. In the present case, sequential therapy with COP + Chi and Lipo-MIT failed to control disease progression, highlighting the challenges of managing R/R AITL in elderly patients.

The aberrant activation of class-I PI3K is a defining feature of cancer (Fruman et al., 2017). The ICOS–PI3K pathway has been identified as the principal mediator of Tfh cell transformation in AITL (Cortes et al., 2018). Furthermore, the tumor microenvironment of AITL is populated by a diverse array of T- and B-cell subsets with aberrant differentiation profiles (Chiba and Sakata-Yanagimoto, 2020). The patient exhibited EBV-DNA positivity in the peripheral blood but EBER negativity in tumor cells. This dissociation highlights the complex role of EBV in AITL, where systemic EBV-driven B-cell activation, evidenced by a high viral load, coexists with the underlying T-cell malignancy. This observation aligns with previous reports indicating that EBV primarily infects microenvironmental B cells rather than the neoplastic T-cell population (Chiba and Sakata-Yanagimoto, 2020). This suggests the importance of EBV testing in the risk stratification and monitoring of potential B-cell clonal expansion. PI3K plays a role in the development and signaling of T and B cells (Okkenhaug and Vanhaesebroeck, 2003). Collectively, targeting PI3K represents a promising avenue for the treatment of R/R AITL. At present, five class-I PI3K inhibitors have been approved by the U.S. Food and Drug Administration, whereas others are undergoing clinical trials (Li et al., 2024). Interestingly, the utilization of PI3K inhibitors for the management of hematological malignancies in the Chinese population has been associated with enhanced response rates compared to those in other populations (Zou et al., 2022).

Linperlisib is a next-generation PI3Kδ inhibitor with mild activity against PI3Kγ. The efficacy of linperlisib has been demonstrated in B-cell lymphomas, especially follicular lymphoma (Jiang et al., 2021; Wang et al., 2023). A phase-Ib clinical trial recently revealed that linperlisib could improve the survival of R/R PTCL. The study enrolled 16 R/R AITL cases, with an ORR of 81%, and eight patients achieved CR (Jin et al., 2024). Moreover, a phase-II study revealed that R/R AITL patients exhibited superior outcomes following treatment with GemOx in combination with epigenetic therapy compared with epigenetic therapy alone. The ORR and CR were 97.1% and 66.7%, respectively, with median PFS and OS of 17.2 and 38.8 months, respectively (Ding et al., 2024). Furthermore, linperlisib exhibited a lower incidence of hepatotoxicity than other PI3K inhibitors, and the GemOx combination therapy was well tolerated (Ding et al., 2024; Hanlon and Brander, 2020; Jiang et al., 2021; Jin et al., 2024). In this elderly patient with R/R AITL, four cycles of linperlisib + GemOx induced sustained PR, followed by ongoing linperlisib monotherapy. The regimen was exceptionally well tolerated with no grade ≥2 toxicities. This case reinforces the therapeutic potential of PI3Kδ inhibition in R/R AITL, particularly in elderly patients who are ineligible for intensive therapies.

In conclusion, we report the case of an elderly patient with AITL who experienced relapse following CHOP therapy, exhibited refractoriness to both chidamide and Lipo-MIT, and ultimately achieved a clinical response to treatment with the PI3Kδ inhibitor linperlisib. Our findings provide critical real-world evidence supporting linperlisib as a safe and effective salvage option for elderly patients with R/R AITL, addressing a significant unmet need in this high-risk population.

## Data Availability

The original contributions presented in the study are included in the article/supplementary material; further inquiries can be directed to the corresponding authors.

## Abbreviations

AITL, angioimmunoblastic T-cell lymphoma; ALB, albumin; ALP, alkaline phosphatase; ALT, alanine aminotransferase; BM, bone marrow; BMMC, bone marrow mononuclear cells; CRP, C-reactive protein; CT, computed tomography; EBER, EBV-encoded RNA; EBV, Epstein–Barr virus; ECOG, Eastern Cooperative Oncology Group; GemOx, gemcitabine and oxaliplatin; GGT, gamma-glutamyl transferase; Hb, hemoglobin; IHC, immunohistochemical; LDH, lactate dehydrogenase; Lipo-MIT, mitoxantrone hydrochloride liposome injection; nTFHL-AI, nodal TFH cell lymphomas angioimmunoblastic-type; ORR, overall response rate; OS, overall survival; PBMC, peripheral blood mononuclear cells; PET–CT, positron-emission tomography–computed tomography; PFS, progression-free survival; PIT, prognostic index for PTCL; PLTs, platelets; PR, partial remission; PTCL, peripheral T-cell lymphoma; R/R, relapsed/refractory; Tfh, follicular helper T; VAFs, variant allele frequencies; WBCs white blood cells.
